# Transcranial Direct Current Stimulation With Halo Sport Enhances Repeated Sprint Cycling and Cognitive Performance

**DOI:** 10.3389/fphys.2019.00118

**Published:** 2019-02-14

**Authors:** Lingyan Huang, Yuqin Deng, Xinyan Zheng, Yu Liu

**Affiliations:** ^1^Key Laboratory of Exercise and Health Sciences of the Ministry of Education, Shanghai University of Sport, Shanghai, China; ^2^School of Sports Science, Nantong University, Nantong, China

**Keywords:** tDCS, Halo Sport, exercise performance, cognition, Stroop test

## Abstract

The present study investigated the effects of transcranial direct current stimulation (tDCS) using the Halo Sport device on repeated sprint cycling ability and on cognitive performance. In this triple-blind, randomized, sham-controlled study, nine physically active participants received either a placebo stimulation (Sham) or real stimulation (Halo) for 20 min. Participants then performed 5 × 6-s sprints interspersed with 24 s of active recovery on a cycle ergometer. Peak and mean power output were measured for each sprint. In addition, cognitive performance in terms of reaction time (RT) and accuracy (ACC) was assessed via Stroop test pre- and post-stimulation. There was a significant interaction for mean power output [*F*(4,32) = 2.98, *P* = 0.03]. A main treatment effect was observed in all of the repeated sprints apart from the initial one. Halo did not affect RT in either the congruent or incongruent condition but did increase ACC in the incongruent condition [*F*(1,8) = 10.56, *P* = 0.012]. These results suggest that tDCS with the Halo Sport system is able to enhance aspects of sprint cycling ability and cognitive performance.

## Introduction

Non-invasive electrical brain stimulation is an emerging technique that claims to improve training effects and boost exercise performance. The rationale for such effects is based on the ability of the stimulation to safely modulate brain excitability and functional plasticity (Angius et al., 2017). The Halo Sport device is a commercial system that consists of a headset similar to conventional headphones. Halo Sport uses transcranial direct current stimulation (tDCS) in which weak direct currents (DC) below 2–3 mA is delivered for a period of minutes over the scalp through surface electrodes, termed primers, with the intention of inducing changes in both sides of the motor cortex.

The primary motor cortex (M1) is a complex network of interconnected localized groups of neurons with similar inputs and outputs, aimed to control movements (Schieber, 2001). The role of the M1 is to generate neural impulses that control the execution of movement (Moscatelli et al., 2016a). It is claimed that Halo Sport produces changes in motor cortex excitability. Therefore, Halo Sport may improve exercise performance. One possible mechanism is that the electrical stimulation induces increases in intracortical facilitation and motor cortex excitability, allowing motor-cortex neurons to build neural connections more easily, enhancing motor drive to the muscles (Hornyak, 2017).

Halo Sport has been used in training and competition, but its effects on physical performance remain elusive. Early studies investigated the effect of tDCS on physical performance using single joint isometric exercise (Cogiamanian et al., 2007). However whole-body exercise better represents real sporting competition than single joint exercise and therefore cycling performance is likely to be more suitable for assessing the ergogenic effect of tDCS. Anodal tDCS applied to M1 of healthy volunteers has been reported to enhance cycling performance (Okano et al., 2015; Vitor-Costa et al., 2015; Angius et al., 2016) and similar effects may be expected for the Halo Sport device. However, no study to date has examined whether Halo Sport applied over the motor cortex is able to enhance cycling performance.

Excellence in sport performance requires not only physical and motor capabilities, but also sensory-cognitive skills (Moscatelli et al., 2016b). Halo Sport is thought to act as a central nervous stimulant, and it may affect cognitive and psychomotor functioning during exercise. To data, no studies have examined the effect of Halo Sport on cognition. Anodal tDCS applied to the dorsolateral prefrontal cortex (DLPC) of healthy volunteers has been reported to enhance the cognition (Dockery et al., 2009; Stone and Tesche, 2009; Zaehle et al., 2011) and similar effects may be expected for the Halo Sport device. Moreover, other studies have reported that tDCS is a central nervous stimulant and has positive effects on cognitive functioning by affecting perception and attention (Shin et al., 2015). This finding could suggest that the performance-enhancing effects of tDCS are due to altered central nervous system function, possibly related to the attenuation of central fatigue effects (Vitor-Costa et al., 2015).

The primary aim of the present study was to examine the effects of Halo Sport on repeated sprint and cognitive performance. It was hypothesized that Halo Sport would improve repeated cycle sprint performance and cognitive function.

## Materials and Methods

### Participants

Participants were deemed eligible using the following criteria: (1) age between 18 and 30 years; (2) males; (3) no diagnosis of neurological, or psychiatric disorders; (4) no history of drug or alcohol abuse; (5) not enrolled in another trial involving weight training; and (6) being physically active (practicing physical activities at least three times a week for at least 6 months; Vitor-Costa et al., 2015). Nine males (age, 20 ± 1.2 years; height, 176.8 ± 6.6 cm; mass, 73.1 ± 6.5 kg) volunteered to participate in the exercise trial. All participants were fully informed of the nature and possible risks of the study before giving written consent. The local ethical committee of Shanghai University of Sport approved the experimental protocol.

### Study Design

**FIGURE 1:**
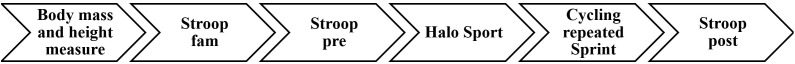
Time line of one experimental trial. Stroop fam, familiarization trials; Stroop pre, Stroop task at baseline; Stroop post, Stroop test after Halo Sport stimulation.

This study was a single blinded, randomized, placebo-controlled, crossover study with a repeated measures design. The subjects visited the laboratory twice. Written informed consent was obtained from all participants before study enrolment. On the day of the experiment, participants were asked to go to the toilet and empty their bladder, then they had their body mass and height measured. Subjects were seated in a comfortable chair for the cognitive tasks (Stroop tasks). Stroop tasks consisted of one practice trial and one baseline (Stroop pre). Following that, participants received a 20-min Halo Sport session either with (Halo) or without (Sham) electrical current delivered to the primers. During stimulation, subjects were seated, closed eyes, kept the same posture and quiet. All subjects received all stimulation conditions. The two experimental trials were separated by 5 days in a counterbalanced order, and conducted at the same time of day to eliminate any effects of circadian variations. At the end of the Halo Sport session, the subjects walked to a cycle simulator and started the exercise protocol. The cycle sprint exercise was based on a previously reported reliable protocol (McGawley and Bishop, 2006). Briefly, after a 5-min warm-up on a calibrated Monark cycle ergometer, the participant was then required to pedal at 50 rpm before being given a verbal countdown to commence a 6-s maximal sprint effort with a resistance of 10% of body mass applied to the front wheel. Five 6-s sprints were completed, with 24 s of unloaded pedaling between each effort. The peak power output and mean power output were recorded in each 6-s loaded sprint. Finally, the Stroop tasks were repeated (Stroop post). The protocol is shown in Figure 1.

### Halo Sport Procedures

Halo Sport is a commercial tDCS device made by© Halo Neuroscience (San Francisco, CA, United States). In addition,© Halo Neuroscience provided permission for their name and equipment to be used in this publication in our study. Halo Sport is designed as a self-contained headset similar in appearance to audio headphones. Three studded foam electrodes termed primers (24 cm^2^/primer), which are wetted prior to use, make the electrical contact with the head. As with normal headphones, Halo Sport needs to be positioned over the vertex of the head. In this position, the primers lie across the top of the head, spanning from ear to ear, with the aim of stimulating both sides of the motor cortex. The electrodes are connected to a continuous current electric stimulator, driven by a Lithium-ion (LiPo) cell (36 V). The maximum energy output was 2.2 mA and was controlled by the Halo application which was set using an Iphone or Ipad.

Participants reclined in a chair, in resting state. The Halo Sport headset was correctly positioned on the head of subjects, and the electrical current was ramped up to 2.0 mA over the course of 30 s. In the active Halo Sport group, the current intensity was maintained at this level for 20 min, whereas in the sham Halo Sport group it was ramped-down after 30 s. This stimulation procedure is similar to that used in previous studies of tDCS (Gandiga et al., 2006).

### Stroop Task

The Stroop test is a classical assessment that measures multiple aspects of cognitive function, including information processing speed, sustaining attention, interference, and inhibition. It is also a neuropsychological assessment that is recommended in research regarding exercise and cognition (Chang et al., 2015). It is sensitive to interference and the ability to suppress an automated response. The Stroop task was programmed and performed on E-prime 1.0 software (Psychology Software Tools, Pittsburgh, PA, United States). This task consisted of two conditions. The congruent condition included three Chinese color words (i.e., 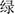 for green, 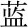 for blue, and 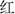 for red) that were displayed in the same color (e.g., “green” displayed in a green font), whereas the incongruent condition included the same three color names but each was displayed in a different color (e.g., “green” displayed in a blue or red font). Subjects had to identify the display color of the word, and the reaction time (RT) and accuracy in doing so was recorded.

Each trial included a fixed cross presented on the center of the screen with 500 ms, followed by a stimulus that was also presented for 500 ms. Participants performed two blocks of 120 trials consisting of congruent trials (trials, *n* = 60) and incongruent trials (trials, *n* = 60) presented in a random order. To avoid the participants’ expectation to stimuli, the interval between the fixed cross and the stimulus presentation was randomly varied between 300 and 800 ms, and the inter-stimulus interval (ISI) was 1500 ms. The RT and accuracy (ACC) were recorded to evaluate Stroop performance. In addition, we used the “interference index” in the Stroop effect as one index to evaluate Stroop performance. The “interference index” was calculated via RT of the incongruent condition minus RT of the congruent condition.

### Statistical Analyses

Statistical analyses were conducted with SPSS v. 20 (IBM, United States). Alterations in peak power output, mean power output, RT, ACC and interference index of Stroop effect were assessed via two-way (treatment × time) repeated-measures ANOVAs. Significant main or interaction effects were followed by appropriate *post hoc* analyses with LSD. Between-stimulation differences in mean peak power output and mean power output were analyzed using a paired-sample *t*-test. The magnitudes of differences in the changes in mean peak power output, in mean power output, and in interference index of Stroop effect between treatments were calculated as Cohen’s effect size (ES). The criteria to interpret the magnitude of ES were as follows: <0.2, trivial; 0.2–0.5, small; 0.5–0.8, moderate; and >0.8, large (Cohen, 1992). Data are presented as mean ± SD. Statistical significance was accepted at *P* < 0.05.

## Results

### Repeated Sprint Ability

#### Peak Power Output

**FIGURE 2:**
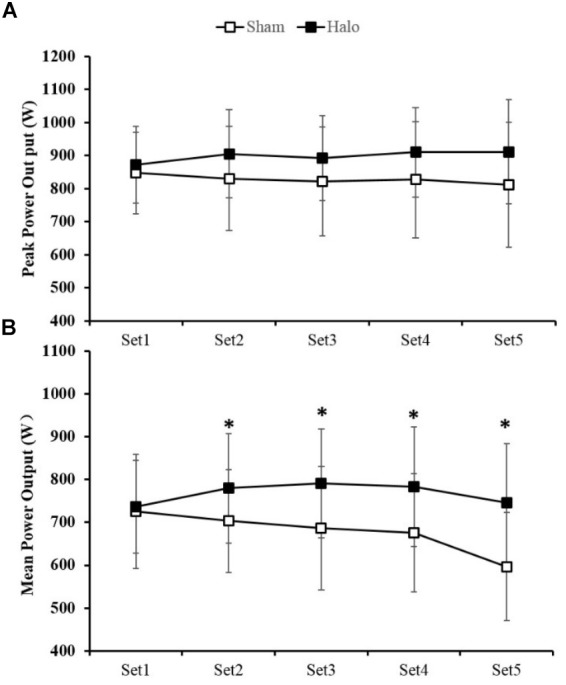
Changes in peak power output **(A)** and mean power output **(B)** for each set of the repeated sprint test (5 set × 6-s sprint with 24-s active recovery) for the conditions of active (Halo) and sham (Sham) Halo Sport. ^∗^Significant treatment effect (*p* < 0.05). Values are mean ± SD.

Figure 2A summarizes changes in peak power every sprint set for each treatment. A 2 × 5 mixed ANOVA revealed that there was no significant interaction for peak power output [*F*(4,32) = 0.91, *P* = 0.47]. A trend for greater mean peak power output following Halo Sport was observed (Sham: 827.8 ± 145.3 W; Halo: 898.3 ± 116.3 W; *P* =0.07). Compared with the Sham group, Halo Sport stimulation showed a moderate effect on mean peak power output (ES = 0.53).

#### Mean Power Output

Figure 2B shows changes in mean power every sprint set for each treatment. A 2 × 5 mixed ANOVA revealed that there was a significant interaction for mean power output [*F*(4,32) = 2.98, *P* = 0.03]. A main treatment effect was observed in Set 2 (Sham: 703.4 ± 128.5 W; Halo: 779.8 ± 128.1 W; *P* < 0.05), 3 (Sham: 686.9 ± 154.5 W; Halo: 791.5 ± 127.4 W; *P* < 0.05), 4 (Sham: 676.1 ± 147.8 W; Halo: 783.5 ± 139.0 W; *P* < 0.05) and 5 (Sham: 596.6 ± 134.8 W; Halo: 745.3 ± 139.1 W; *P* < 0.05). Compared with Sham group, Halo Sport stimulation showed a moderate effect on mean power output (ES = 0.60).

### Stroop Test

#### Reaction Time

For the incongruent condition, a 2 × 2 mixed ANOVA revealed that there was a significant main effect of RT [*F*(1,8) = 17.68, *P* = 0.003, Table 1], with shorter RTs after stimulation. However, main effects for treatment and the interaction of treatment by time were not significant [*F*(1,8) = 0.047, *P* = 0.83]. For the congruent condition, we also found a significant main effect of RT, *F*(1,8) = 5.69, *P* = 0.04, with, again, shorter times observed in the post-stimulation test. However, main effects for treatment and the interaction of treatment by time were not significant, *F*(1,8) = 0.48, *P* = 0.51 (Table 1).

**Table 1:** The reaction time, interference index, and accuracy rate of the Stroop test.

		Sham group		Halo group	
		Pre	Post	Pre	Post
Reaction time	Incongruent (ms)	636.78 ± 54.65	602.21 ± 51.04^#^	652.96 ± 72.18	613.65 ± 65.81^#^
	Congruent (ms)	581.21 ± 21.78	568.39 ± 28.78^#^	586.53 ± 32.17	565.35 ± 39.73^#^
Interference index (ms)		38.89 ± 43.95	33.82 ± 30.64	66.43 ± 48.95	48.30 ± 33.14
Accuracy rate	Incongruent	0.91 ± 0.05	0.88 ± 0.06^#^	0.87 ± 0.07	0.92 ± 0.05^#^∗^^
	Congruent	0.95 ± 0.03	0.95 ± 0.03	0.93 ± 0.05	0.96 ± 0.02^#^


*Data are presented as mean ± SD. Pre, pre-stimulation; Post, post-stimulation; ^∗^, significant difference with Sham, ^#^, significant difference with Pre.*

#### Accuracy

For the incongruent condition, a 2 × 2 mixed ANOVA revealed that there was a significant interaction for ACC [*F*(1,8) = 10.56, *P* = 0.01, Table 1]. ACC was significantly decreased after stimulation in the Sham group (Pre: 0.91 ± 0.05; Post: 0.88 ± 0.06; *P* < 0.05). In the Halo group, ACC was significantly increased after stimulation (Pre: 0.87 ± 0.07; Post: 0.92 ± 0.05; *P* < 0.05). However, for the congruent condition, a 2 × 2 mixed ANOVA revealed that there was a significant main effect of ACC (*F* = 9.59, *P* = 0.015, Table 1), where an increase in ACC was observed in the post-stimulation test. However, main effects for treatment and the interaction of treatment by time were not significant, *F*(1,8) = 0.96, *P* = 0.36.

### Stroop Effect

With respect to the “interference index” in the Stroop effect, no significant interaction was found (Table 1). Compared with pre-stimulation, Sham showed a trivial effect (ES = 0.13), and Halo showed a small effect (ES = 0.43).

## Discussion

This is a novel study to show the effects of tDCS using the Halo Sport device on repeated sprint cycling ability and on cognitive performance. We found that tDCS with the Halo Sport device improved repeated sprint cycling power output and Stroop performance.

Interest in the possible ergogenic effect of non-invasive brain stimulation is growing and whilst there are a number of studies looking at tDCS there are few reports specifically concerning the Halo Sport device. Early studies investigated the effect of tDCS on physical performance using single joint isometric exercise (Cogiamanian et al., 2007). However whole-body exercise better represents real sporting competition than single joint exercise and therefore cycling performance is likely to be more suitable for assessing the ergogenic effect of tDCS. Of those studies that have examined the effect of tDCS on physical performance in cycling, the evidence is inconsistent (Angius et al., 2015, 2016; Okano et al., 2015; Vitor-Costa et al., 2015; Barwood et al., 2016). Okano et al. (2015) reported that 2 mA for 20 min of anodal tDCS targeting the temporal cortex enhanced maximal power output by about 4%. On the other hand, using similar methodology as Okano et al. (2015); Barwood et al. (2016) observed that following 20-min of anodal tDCS at 1.5 mA over the left temporal cortex, 20 km cycling time trial performance was unaffected. In addition, they also found no effect of 20-min of tDCS at 2.0 mA on exercise performance in the heat. Such inconsistencies indicate that the effects of tDCS may be dependent on a range of factors including experimental environment, stimulation duration and intensity, and electrode configuration and position on the head. The Halo Sport device is one commercial form of tDCS and any effects it produces may be affected by such factors (Angius et al., 2017).

The present study is the first to provide evidence that Halo Sport is able to improve cycling performance. We found that 20 min of stimulation at 2mA with Halo Sport significantly enhanced the mean power output during cycling sprints. In previous work, it has been reported that 2 mA of stimulation for 20 min targeting the motor cortex bilaterally of tDCS enhanced muscle power in lower limb exercise (Lattari et al., 2017). Therefore, the ability of Halo Sport to enhance cycling performance may be related to the increases in lower limb muscle power during cycling. The precise mechanism through which Halo Sport improves exercise performance is unknown. Previous studies suggested that the performance-enhancing effects of tDCS are due to altered central nervous system function, possibly related to the attenuation of central fatigue effects (Vitor-Costa et al., 2015). In the present study, we observed that Halo Sport was able to improve cognitive test. Cognitive decrease is related to central fatigue (Meeusen, 2014), therefore our finding indirectly evidence that the ability of Halo Sport to enhance cycling performance may be related to inhibit central fatigue. One possible mechanism is that the electrical stimulation induces increases in intracortical facilitation and motor cortex excitability, allowing motor-cortex neurons to build neural connections more easily, enhancing motor drive to the muscles, increasing power output of cycle and metal performance, improving cycling performance (Hornyak, 2017).

Moreover, Tanaka and Watanabe (2012) developed a neural circuit for the action of this facilitatory pathway. First, sensory input from the peripheral system to M1 reduces motor output (supraspinal fatigue), and a neural pathway that interconnects the spinal cord, thalamus, secondary somatosensory cortex, medial insular cortex, posterior cingulate cortex, anterior cingulate cortex, premotor area, supplementary motor area (SMA), and primary motor cortex constitutes the inhibition system. Then, a facilitation system increases motor output from M1 to overcome the existing supraspinal fatigue. A re-entrant neural circuit that bridges the limbic system, basal ganglia, thalamus, orbitofrontal cortex, prefrontal cortex, anterior cingulate cortex, premotor area, SMA, and primary motor area represents the facilitation system. Motivational input to this system enhances SMA activity, and subsequently, motor cortex enhances motor output to the peripheral system (Vitor-Costa et al., 2015). Thus, the output (exit of information from the motor cortex to the corticospinal pathways and, consequently, motoneurons) from M1 is regulated primarily by the balance between inhibition and facilitation, leading us to speculate that Halo Sport has a facilitatory effect for increasing power output of cycling. This hypothesis needs to be evaluated in future studies.

To our knowledge, this is the first study to observe that Halo Sport can enhance cognitive performance. It is difficult to compare our findings with those of previous non-invasive neuromodulation studies on cognitive function. Anodal tDCS applied to the DLPC of healthy volunteers has been reported to enhance the executive function of cognition (Dockery et al., 2009; Stone and Tesche, 2009; Zaehle et al., 2011). The executive function is one aspect of cognition, and it generally consists of mental-set-shifting, information updating, and inhibition of prepotent responses (Miyake et al., 2000; Hofmann et al., 2012). Fregni et al. (2005) reported that anodal tDCS of the prefrontal cortex enhanced ACC of 3-back which is a test for information updating performance. They proposed that tDCS could improve the information updating performance aspect of executive function. In the present study, we found similar results to those of Fregni et al. (2005). Following Halo Sport stimulation over both sides of the motor cortex, the ACC enhancement of Stroop incongruent trial in the Halo group cannot be accounted for by slowed responses, as response times were not changed by stimulation. These results showed that Halo Sport stimulation leads to an enhancement of Stroop performance. Stroop test is a classic task for inhibition of prepotent responses. Therefore, Halo Sport has a positive effect on executive function. These results indicate that Halo Sport may be useful for enhancing all types of exercise in which concentration, RTs, and technical/tactical skills have a major influence on both physical and mental performance, such as cycling/mountain biking, skiing, most ball game and so on.

In the present study, we have only shown the positive effect of Halo Sport on Stroop performance, but the mechanisms behind this phenomenon are unknown. Milham et al. (2003) reported that during the Stroop task, the DLPC is the primary region involved in the implementation of top down attention control. Additionally, according to Krompinger and Simons (2011) the DLPC resolves conflicts that occur during information processing of incongruent stimuli during the Stroop task. Therefore, the Stroop performance is related to activation of the DLPC. Moreover, previous work indicates that bilateral stimulation of the motor cortex induces widespread changes in functional connectivity, in particular with the prefrontal cortex, and the primary and secondary motor cortices (Sehm et al., 2012). Anatomically, M1 is located next to the SMA. The activation of M1 might affect the active SMA, whose functions are considered to plan the movement and make the decision about when to start an action (Nachev et al., 2008; Coull et al., 2016). And previous studies have shown that SMA might work with dorsal anterior cingulate cortex (dACC) to process the cognitive interference, which is produced by conflict conditions of Stroop task (Liu et al., 2004; Deng et al., 2018). DACC sends the signal on cognitive interference to DLPFC, which would participate in resolving the cognitive interference (MacDonald et al., 2000; Liu et al., 2004). In the present study, the improved Stroop performance may be due to Halo Sport increasing the activation of the DLPC in addition to both sides of the motor cortex. Further studies are needed to clarify the effects of Halo Sport on brain activity.

## Conclusion

tDCS with the Halo Sport device improved repeated sprint cycling power output and Stroop performance. These results indicate that Halo Sport may have the potential to enhance performance across a wide range of exercise activities that entail both physical and cognitive demands.

## Ethics Statement

This study was carried out in accordance with the recommendations of human experimental guidelines, the local ethical committee of Shanghai University of Sport. All participants were fully informed of the nature and possible risks of the study before giving written consent. The local ethical committee of Shanghai University of Sport approved the experimental protocol.

## Author Contributions

XZ and LH conceived the study. YL supervised the study. XZ and YD designed the experiments. XZ and LH carried out the experiments. XZ and YD analyzed the data. XZ and LH wrote the manuscript. All authors approved the final version of the submitted manuscript.

## Conflict of Interest Statement

The authors declare that the research was conducted in the absence of any commercial or financial relationships that could be construed as a potential conflict of interest.
